# Troubleshooting Public Data Archiving: Suggestions to Increase Participation

**DOI:** 10.1371/journal.pbio.1001779

**Published:** 2014-01-28

**Authors:** Dominique G. Roche, Robert Lanfear, Sandra A. Binning, Tonya M. Haff, Lisa E. Schwanz, Kristal E. Cain, Hanna Kokko, Michael D. Jennions, Loeske E. B. Kruuk

**Affiliations:** 1 Division of Evolution, Ecology and Genetics, Research School of Biology, Australian National University, Canberra, Australian Capital Territory, Australia; 2 Australian Research Council Centre of Excellence for Coral Reef Studies, Australian National University, Canberra, Australian Capital Territory, Australia; 3 Institute of Evolutionary Biology, University of Edinburgh, Edinburgh, United Kingdom; University of California Davis, United States of America

## Abstract

An increasing number of publishers and funding agencies require public data archiving (PDA) in open-access databases. PDA has obvious group benefits for the scientific community, but many researchers are reluctant to share their data publicly because of real or perceived individual costs. Improving participation in PDA will require lowering costs and/or increasing benefits for primary data collectors. Small, simple changes can enhance existing measures to ensure that more scientific data are properly archived and made publicly available: (1) facilitate more flexible embargoes on archived data, (2) encourage communication between data generators and re-users, (3) disclose data re-use ethics, and (4) encourage increased recognition of publicly archived data.

## Abstract 2

Public data archiving has many benefits for society, but some scientists are reluctant to share their data. This Perspective offers some practical solutions to reduce costs and increase benefits for individual researchers.

Good science relies on transparent, reproducible results, and scientific data are often collected with public funds [1]–[3]. For these reasons, funding agencies, publishers, and researchers are increasingly encouraging public data archiving (PDA) into open-access databases [1]–[8]. It is widely accepted that the benefits of PDA to the scientific community greatly outweigh the costs [6]–[10]. However, decisions to archive data are currently made by individual researchers, and it is less obvious that the benefits of PDA outweigh the costs for all individuals [10]. This probably explains why PDA is far from universal in the biological sciences (e.g., [11],[12], but see major initiatives in genomics [13]), and why many researchers still harbour concerns about making their data publicly available [10],[14]–[17]. This is particularly true in fields such as ecology and evolutionary biology, where datasets are often complex, have a long shelf life, and can be used to test multiple hypotheses [3],[7],[18] (Figure 1). The benefits of data sharing have been extensively discussed [1],[3],[5],[7],[10],[19], but the real and perceived costs have received far less attention in the literature. Acknowledging and discussing how to ameliorate these costs is critical to promoting PDA in all disciplines. Here, we hope to stimulate discussion by briefly reviewing the costs and benefits of PDA and suggesting practical solutions to reduce the costs and increase the benefits for individual researchers.

**Figure 1:**
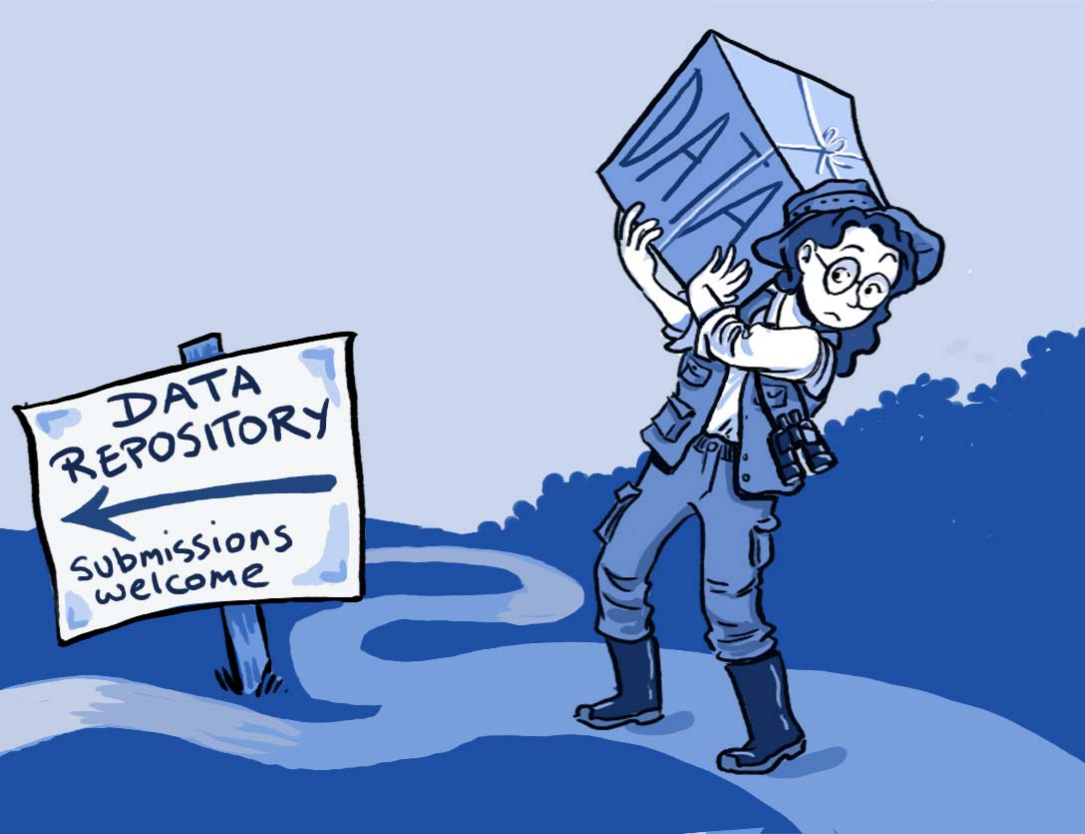
Researchers can be reluctant to share their data publicly because of real and/or perceived individual costs. *Illustration credit: Ainsley Seago.*

The value of PDA can be viewed either from the perspective of the scientific and broader community as a whole (group), or from that of individual researchers. Group benefits are substantial and have driven the formulation of policies aimed at establishing a culture of data archiving and sharing. PDA increases data preservation by avoiding losses from hardware malfunction or obsolescence [7], or from researchers moving on to different projects or retiring. PDA also encourages good metadata production to ensure that datasets are interpretable [8]. In turn, open access to data increases the ability to evaluate and reproduce studies [1],[9],[10], encourages a stronger sharing culture [5], improves the return per research dollar [10],[19], and increases opportunities for teaching and learning [7],[10]. Currently, group costs include the financial costs of maintaining public databases such as figshare, Dryad, TreeBASE, and GenBank [7],[20]. Potential future costs might arise if large amounts of freely available primary data online lead to the publication of misinterpretations of datasets, which is more likely when the intricacies of data collection and biological considerations are difficult to convey in metadata files [21]. Additionally, spurious conclusions may arise because of type I errors from data dredging (i.e., exploratory analyses) and subsequent publication bias [22]. Finally, if data re-use has perceived advantages over collecting primary data for individual researchers (see below), this could decrease the overall amount of primary data collected and potentially create long-term group costs.

At the individual level, there are various benefits to PDA for researchers who collect primary data. These include increased citation of the original study and/or of the archived datasets [7],[23], recognition through metrics such as “altmetrics” [24] and the proposed new Data Usage Index [25] and “data deposition” metric [16], potential co-authorship of new studies [7], improved data management requirements (which ultimately make it easier for researchers to re-use their own data) [7],[10], and prizes for pursuing “open science” initiatives (e.g., the ASAP award, http://asap.plos.org). Individual costs include the time required to generate appropriate metadata and data descriptors to facilitate re-use [7],[9], the modest financial costs of submitting data to some archives [26], and the need to monitor how one's data are used (e.g., [27],[28]) because of concerns regarding misinterpretation of data by researchers with less experience with the study system [29]. In our experience, however, individuals are most concerned about the loss of priority access following PDA, which could generate competition with others when conducting subsequent analyses (see [3],[16],[17],[30]). Many individuals judge that the benefits of PDA, such as an increased citation rate for an initial paper [31], will not compensate for the future publications lost by renouncing priority access to the data they collected [32]—the fear of being “scooped”. Given intense competition for grants and academic positions, where publications are the major currency for assessing performance [20],[21], it is rational for an individual to make decisions that primarily maximise his/her publication rate rather than maximising the benefits for science at large [20],[32], and there is therefore substantial risk of these concerns affecting rates of PDA.

Many journals and funding agencies (e.g., the National Science Foundation [US], the National Institutes of Health [US], the Natural Environment Research Council [UK]) now require PDA following publication [7],[33]—for specific policies of journals and funding agencies see [33]–[35]. This requirement provides an effective “stick” [36], but authors who are concerned about PDA can simply avoid these journals, or can archive data in a way that makes them difficult to re-use. Currently, most journals do not police the quality of archived data [36],[37], making it easy to circumvent the system if desired (e.g., by not archiving data at all or by archiving either incomplete data or data in inappropriate formats) [16],[17],[38]. Unfortunately, in biology, the concerns regarding PDA are possibly strongest for large-scale studies conducted over multiple geographic locations, seasons, or years, which require substantial financial and logistic resources (e.g., those in ecology, evolutionary biology, and climate change science). These datasets may be vital for elucidating trends in species distributions, phylogenetic relationships, or selection pressures through time, as well as the wider effects of climate change, habitat loss, and invasive species [18],[39]. Where such data involve large teams of researchers, additional concerns arise as to overlap of data re-users' activities with ongoing work, particularly by graduate students. PDA of these data is costly for authors in a system that requires rapid release into the public domain (e.g., figshare offers no embargo option), making it difficult for the original authors to reap sufficient rewards (i.e., publications) for their substantial initial investment in data collection. Consequently, many valuable datasets are improperly archived or not archived at all (see [16],[38]), and therefore never enter the public domain.

A slight shift in the protocols for the use of public data could complement existing measures to promote PDA by lowering costs and increasing benefits for individual data collectors. In essence, more (or larger) “carrots”, not “sticks”, are needed to increase participation in PDA [40]. Our proposed measures are four-fold: (1) facilitate more flexible data embargoes, (2) encourage better communication between data re-users and data collectors, (3) disclose data re-use ethics, and (4) encourage the recognition of publicly archived datasets by academics, funding bodies, and hiring committees.

## Facilitate More Flexible Embargoes on Archived Data

**Figure 2:**
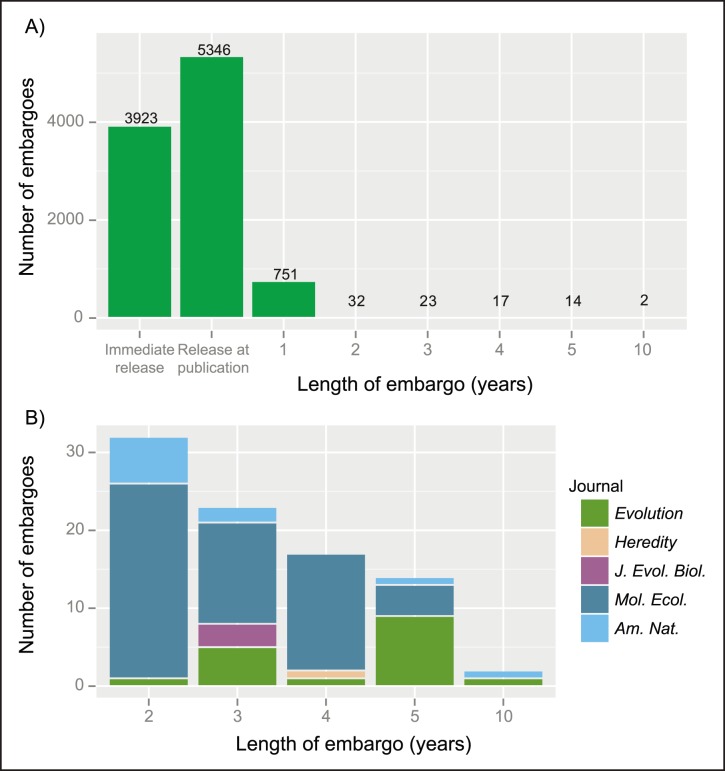
Embargoes chosen by Dryad data authors. (A) Embargo selections of Dryad data authors for the 10,108 files in Dryad (http://datadryad.org/) deposited from inception to September 20, 2013. Data include only datasets related to articles published in journals for which the authors had the option of selecting an embargo. (B) Long-term embargoes (>1 year) by journal that granted them. Data were obtained from [42].

By default, public repositories release archived datasets when an article is published [7],[8]. However, in adopting the Joint Data Archiving Policy (JDAP) [33], the American Genetic Association (which publishes the *Journal of Heredity*) emphasised the importance of the “right of first use” by data providers, given the substantial investments of individual researchers in generating and curating datasets [41]. This right can be facilitated by embargoing data for a certain period. The question then becomes: how long is a reasonable embargo? Some journals that follow the JDAP allow data to be placed under embargo for up to a year [8],[21]. For example, 7.4% of authors that archived data in Dryad prior to September 2013 chose a one-year no-questions-asked embargo when this option was available (Figure 2) [42]. Longer embargoes can be obtained upon appeal to editors, but currently, anything longer than one year requires special agreement. A recent analysis of re-use of gene expression data suggested that a two-year embargo is sufficient to outlive most re-uses of published data by the original authors [31]. Arguably, however, this time frame is too short for many subdisciplines of ecology and evolution (e.g., with field data collected across multiple years and datasets with multiple potential uses), where data less often become obsolete due to new technologies, and where records collected years or decades previously may still be re-used (e.g., [43]). In such cases, embargoes of up to five years may be more appropriate to allow data generators sufficient time to use the data fully for their planned purpose. Examples could include when a project involves an extensive period of data collection followed by, or concurrent with, analysis and publication of several aspects of the data; when the data collectors intend to extend a dataset to include additional species, seasons, years, etc.; when the data constitute a significant portion of a student's dissertation; and situations such as interruption of research due to parental or sick leave. Readily granting embargoes of up to five years in such cases could reduce the motivation for avoiding proper archiving of complete datasets, and thereby increase participation in PDA.

To assess current policies on embargoes in data archiving, we conducted an informal survey of journals that follow the JDAP [44]. Of the 33 journals contacted, 21 responded. All but one indicated that requests for extended embargoes are currently rare: authors ask for embargoes exceeding one year in less than 1% of cases. The opinion of editors on extended embargoes varied. Four cited “sensitive” data as the only reason for embargo extensions (e.g., endangered species locations, commercial clauses, human subject data); one journal, according to the editor, requires authors to seek approval from funding agencies before the journal grants extended embargoes. Three journals had very positive views towards extending embargoes, for example, stating that any reason authors make is a good one; only one journal had a formal policy on extending embargoes up to five years when such embargoes supported PhD research, long-term datasets, etc. Overall, the editors who responded to our survey were receptive to longer embargoes where sufficient justification could be given. Requesting longer embargoes could therefore ease one of the most significant concerns regarding PDA: having priority access to data for sufficient time to generate additional publications using the same data.

Offering longer embargoes need not impede data sharing if most authors continue to opt for shorter or no embargoes (Figure 2). Authors opting for a longer embargo period could be required to release metadata, with encouragement for interested data re-users to contact them directly to request access to datasets prior to the embargo expiry (see the next section). The TRY Plant Trait Database is an excellent example of how metadata can facilitate data sharing of private or embargoed data (http://www.try-db.org). Clearly, open data are preferable to embargoed data, but properly archived, searchable data under a temporary embargo are better than un-archived data that will never become open.

## Encourage Communication between Data Generators and Re-Users

We need to encourage a culture of, and an agreed-upon etiquette for, communication between data collectors and data re-users. In a recent case, an unfortunate situation arose in which sequences placed in the Global Initiative on Sharing Avian Influenza Data (GISAID) database were unwittingly used before the original researchers had submitted their own paper. Fortunately, the problem was rapidly resolved by open and reasonable discourse [45]. Basic etiquette and open communication also help to avoid duplicated effort between data collectors and re-users. Of equal importance, good communication reduces the risk of alternative interpretations of data being published by researchers with widely different degrees of knowledge of the study system. This concern is particularly relevant for extensive datasets from complex ecological systems (e.g., [27],[28]). Good communication also has the mutually advantageous benefit that it often facilitates new collaborations: most data collectors are likely to be pleased to hear suggestions for novel ways to use their hard-earned data.

Good communication is the responsibility of all parties, and sensible guidelines have been proposed. White et al. suggest nine simple ways to facilitate data re-use by making data understandable, easy to analyse, and readily available [46]. If data collectors wish to be informed of further uses of their archived data, a request to be contacted should be included with the archived files. Those re-using data are also encouraged to offer co-authorship of any resulting papers if the data provide a “non-trivial” input to the new project [7]. Arguably, data that have been carefully collected, managed, and archived are themselves a “non-trivial” contribution if they constitute a sizable portion of the data used for a publication. However, offering co-authorship will obviously be challenging in many cases—especially if the original study has multiple authors, or if a dataset integrates pre-existing data [21]. Clearly, there is a need for consensus ethical rules for co-authorship attribution when an analysis uses data from multiple studies (e.g., a meta-analysis or synthesis article) [47]. Further discussion is required to establish workable guidelines [21],[45], but in principle, the problems are no more intractable than many that arise over authorship of primary data papers (see [48]). As a useful starting point, Duke and Porter suggest four criteria that must be met for data providers to merit co-authorship: the data are integral to the analysis, the data are novel or unique, the data provider is willing to share authorship, and the data provider is able to participate [21].

## Disclose Data Re-Use Ethics

Ultimately, measures that reduce conflict among parties early on in the data sharing process will promote PDA. Publishers have a key role to play in establishing cultural norms for data re-use [4],[7]. One measure is to require ethical statements about data re-use. Many journals currently require statements about author contributions, conflicts of interest, and animal ethics approval. Journals could similarly require disclosure of the details of data re-use: a brief summary of any effort made to contact the primary researchers, their response, and any discussion about results, interpretation, co-authorship, and consent of re-use of any data under embargo. Journal editors could also consider offering data generators the option to review any paper using their data or to publish a response, with these policies being clear to data re-users on submission of a paper. Similar procedures could apply to grant applications to funding agencies.

## Encourage Increased Recognition of Publicly Archived Data

Following any embargo period, archived datasets generally enter the public domain under the Creative Commons Zero license [49]. The Creative Commons Zero license does not legally require data to be cited when re-used [50]. Adequate recognition of PDA therefore relies on scientific ethics and good practice—citing open datasets is one of the best ways to reward their publication and encourage participation in PDA. Journals can directly contribute to this if their instructions to authors require citing both the dataset and the original article in studies that use publicly accessible data. For example, phylogenetic studies using sequence data from GenBank are encouraged to cite originating papers in addition to accession numbers [16]. In practice, this is challenging because journals often restrict reference lists, and references in supplementary information are not indexed by the main citation services. Because of this, we reiterate a recent call for citation services to recognise references in supplementary information [51].

Ultimately, encouraging funding bodies and employers to recognise data-use metrics will be fundamental to increasing individual-level incentives for PDA. Reassuringly, some funding bodies already have policies that recognise “altmetrics” [52] and research outputs such as datasets, software, code, and patents [24]. Recognition of publicly archived datasets would also be enhanced if academics routinely included information about their published datasets in their curriculum vitae. This effort will be helped by recent initiatives such as ORCID (http://orcid.org/), which collects information on publicly archived datasets in the figshare database (http://figshare.com/). Integration of data from other repositories such as Dryad and GenBank would facilitate quantification of the impact of each researcher's publicly archived data. Importantly, the recent San Francisco Declaration on Research Assessment makes key recommendations for improving the way individual scientist's research outputs, including datasets, are evaluated [53].

In conclusion, the trend towards PDA and greater data sharing has many benefits, but it also generates tensions. Meaningful solutions require frank acknowledgment of the potential differences between the interests of individual researchers and those of the broader scientific community. We hope that researchers, publishers, and database managers will consider these issues when deciding on the best practices for PDA.

## Abbreviations

JDAP: Joint Data Archiving Policy
PDA: public data archiving
